# Real-life data of abiraterone acetate and enzalutamide treatment in post-chemotherapy metastatic castration-resistant prostate cancer in Poland

**DOI:** 10.3389/fonc.2023.1108937

**Published:** 2023-04-03

**Authors:** Dawid Sigorski, Michał Wilk, Angelika Gawlik-Urban, Agata Sałek-Zań, Joanna Kiszka, Mateusz Malik, Katarzyna Czerko, Kamil Kuć, Cezary Szczylik, Tomasz Kubiatowski, Bożena Cybulska-Stopa, Emilia Filipczyk-Cisarż, Lubomir Bodnar, Iwona Skoneczna

**Affiliations:** ^1^ Department of Oncology, Collegium Medicum, University of Warmia and Mazury, Olsztyn, Poland; ^2^ Department of Oncology and Immuno-Oncology, Warmian-Masurian Cancer Center of the Ministry of the Interior and Administration Hospital, Olsztyn, Poland; ^3^ Department of Oncology, Centre of Postgraduate Medical Education, European Health Centre, Otwock, Poland; ^4^ Department of Clinical Oncology, Maria Sklodowska-Curie National Research Institute of Oncology, Kraków, Poland; ^5^ Faculty of Health Sciences, University of Applied Sciences in Tarnów, Tarnów, Poland; ^6^ Department of Clinical Oncology, Subcarpathian Cancer Center, Brzozów, Poland; ^7^ Department of Clinical Oncology, Lower Silesian Oncology, Pulmonology and Hematology Centre, Wroclaw, Poland; ^8^ Department of Clinical Oncology and Radiotherapy, St. John Paul II Mazovia Regional Hospital in Siedlce, Siedlce, Poland; ^9^ Department of Oncology, St. Pio’s Provincial Hospital, Przemyśl, Poland; ^10^ Faculty of Medical and Health Sciences, University of Natural Sciences and Humanities, Siedlce, Poland; ^11^ Department of Oncology, Grochowski Hospital, Warsaw, Poland; ^12^ Cancer & Cardio-Oncology Diagnostics, Maria Sklodowska-Curie National Research Institute of Oncology, Warsaw, Poland

**Keywords:** real-word study, abiraterone acetate, enzalutamide, metastatic prostate cancer, targeted therapy

## Abstract

**Background:**

Abiraterone acetate (ABI) and Enzalutamide (ENZA) are second-generation hormone drugs that show breakthrough activity in post-chemotherapy, metastatic castration-resistant prostate cancer (mCRPC). The leading oncological and urological guidelines indicate both drugs with the same strong recommendation. There is a lack of randomized trials which compare the efficacy of ABI and ENZA. The current study aimed to compare the effectiveness of the drugs with an analysis of prognostic factors related to those drugs.

**Patients and methods:**

The study included 420 patients with docetaxel (DXL) pretreated mCRPC from seven Polish cancer centers. Patients were treated according to inclusion and exclusion criteria in the Polish national drug program (1000 mg ABI and 10 mg prednisone, *n*=76.2%; ENZA, 160 mg; *n*=23.8%). The study retrospectively analyzed the overall survival (OS), time to treatment failure (TTF), PSA 50% decline rate (PSA 50%) and selected clinic-pathological data.

**Results:**

In the study group, the median OS was 17 months (95% CI: 15.6-18.3). The median OS (26.1 vs. 15.7 mo.; *p*<0.001), TTF (14.2 vs. 7.6 mo.; *p*<0.001) and PSA 50% (87.5 vs. 56%; *p*<0.001) were higher in ENZA than in ABI treatment. Multivariate analysis shows that ENZA treatment and PSA nadir <17.35 ng/mL during or after DXL treatment were related to longer TTF. ENZA treatment, DXL dose ≥750 mg, PSA nadir <17.35 ng/mL during or after DXL treatment was related to longer OS.

**Conclusions:**

ENZA treatment may be related to more favorable oncological outcomes than ABI treatment in the studied Polish population of patients. A 50% decline in PSA is an indicator of longer TTF and OS. Due to the non-randomized and retrospective nature of the analysis, the current results require prospective validation.

## 1 Introduction

Prostate cancer (PCa) is one of the most common cancers worldwide. According to the Surveillance Epidemiology and End Results Program, it is estimated that in 2022 as many as 34,500 people will die of PCa (1, 2). Androgen deprivation therapy (ADT) remains the key systemic therapy for patients with metastatic prostate cancer. Despite the initial sensitivity to ADT, PCa transforms into the uncurable castration-resistant stage of the disease (3). Currently registered treatment options for PCa patients consist of chemotherapy (docetaxel (DXL), cabazitaxel) and second-generation antiandrogens (abiraterone acetate (ABI), enzalutamide (ENZA), apalutamide, darolutamide), radiopharmaceutical therapy (Radium-223), immunotherapy (sipuleucel-T, pembrolizumab) and PARP inhibitors (olaparib, rucaparib) (4, 5). The drug selection depends on the castration status, tumor stage and genetic mutation status. According to National Comprehensive Cancer Network, oncological guidelines ABI and ENZA are the first-choice treatment options for patients who progressed after prior DXL chemotherapy and were not treated with novel hormone therapy (category 1) (6).

Advances in cancer pathobiology and understanding the mechanisms contributing to cancer progression allow for designing antiandrogen-targeted therapy. ABI and ENZA changed the treatment landscape for patients with PCa (7, 8). Several differences between these drugs include mechanisms of action, dosing method and pattern of side effects. ABI inhibits 17α-hydroxylase/C17.20-lyase (CYP17), an enzyme involved in the biosynthesis of androgens (8). ENZA inhibits the androgen receptor signaling pathway affecting androgen binding to androgen receptors, translocation of androgen receptors to the nucleus and interaction with DNA (7). Both drugs are used once daily, ABI needs additional steroid supplementation. ABI and ENZA are registered treatment options for chemotherapy-naive and chemotherapy-pretreated PCa patients with metastases. COU-AA-301 and AFFIRM were the phase III randomized clinical trials which showed that ABI and ENZA increase overall survival (OS) in PCa patients previously treated with chemotherapy (7, 8). There is a lack of established predictive factors and head-to-head comparative studies, which may facilitate clinicians’ choice between these two drugs. The current study aimed to compare the oncological outcome measures and evaluate prognostic factors affecting survival in real-life populations of patients treated with ABI and ENZA in Poland.

## 2 Patients and methods

This retrospective analysis was approved by the local bioethics committee in Olsztyn (5/21/VII.) and was conducted in accordance with the Declaration of Helsinki. The analyzed data were obtained from the Polish National Health Fund Drug Program database. The study population consisted of patients with metastatic castration-resistant prostate cancer (mCRPC) receiving 1,000 mg ABI with 10 mg prednisone or 160 mg ENZA once a day who progressed after chemotherapy with DXL and were qualified for this treatment in seven comprehensive cancer centers in Poland (Olsztyn, Otwock, Cracow, Brzozów, Wrocław, Siedlce, Przemysl) between 2014-2021 (ABI) and 2018-2021 (ENZA). The inclusion and exclusion criteria were based on the Polish National Health Fund Department, which reimbursed the treatment. The criteria are mostly equivalent to those from clinical trials.

Inclusion criteria included: age over 18 years, a pathological diagnosis of prostate adenocarcinoma with radiologic evidence of metastases, and a serum testosterone level of 50 ng/dL or less (≤1.7 nmol/L) due to ADT (surgical or pharmacological). All qualified patients received DXL before ABI or ENZA treatment. The disease progression after or during chemotherapy was defined as biochemical progression if a patient had two consecutive increases in the prostate-specific antigen (PSA) concentration (from the lowest PSA level reached during or after DXL) or radiological progression (radiographic evidence of disease progression in bone or soft tissue). All patients had an Eastern Cooperative Oncology Group (ECOG) performance status score of 0 or 1. Patients could not be qualified if they had significant hepatic dysfunction, unstable or uncontrolled cardiovascular disorders, or a history of prior abiraterone acetate, enzalutamide or ketoconazole therapy. All the patients’ characteristics were registered before the initiation of ABI/ENZA treatment.

The primary endpoints of the analysis were:

- The OS, defined as the time between the start of treatment and death from any cause;
- The time to treatment failure (TTF) is described as the time between the initiation of ABI/ENZA to the moment of its termination (due to cancer progression*, unaccepted toxicity, hypersensitivity to the drug or the patient’s death);
- PSA 50% rate - patients with PSA decline 50% or more during the treatment.

Disease progression* was defined according to the Polish drug program:

- The occurrence of at least two of the following three types of progression in total:
  1) Clinical - defined as pain progression (inclusion of a new opioid for more than two weeks) or the occurrence of skeletal-related events or ECOG ≥2 (according to the WHO classification).
  2) Biochemical- defined as PSA progression (three consecutive increases in PSA, measured at least in weekly intervals, with proven increases of at least 50% from ABI/ENZA baseline).
  3) Radiological - the appearance of at least two new metastatic lesions confirmed by bone scan.
- Response Evaluation Criteria In Solid Tumors ver. 1.1 were met (regardless of other types of progression mentioned above).

The analyzed clinicopathological data included:

- Characteristics of PCa: date of diagnosis, Gleason score (GS), primary stage at PCa diagnosis (non-metastatic (M0) vs. metastatic (M1)), sites of metastases at the beginning of ABI/ENZA (bone, only extra bone, both localizations).
- History of PCa treatment: treatment approach at PCa diagnosis (radical vs. palliative), type of castration (LHRH agonists, LHRH antagonists, surgical), the timing of DXL (for hormone-sensitive PCa vs. mCRPC), total administered dose of DXL, duration of chemotherapy, PSA nadir during or after DXL treatment, subsequent lines after ABI/ENZA treatment, type of progression at ABI/ENZA treatment initiation (biochemical, radiological or both).
- Patient characteristics: age, ECOG and body mass index (BMI).

The statistically significant variables in univariate analysis were chosen for multivariate analysis.

Statistical analyses were performed using Stata^®^ Software ver. 14.1 (StataCorp LLC). Nominal parameters were presented as a percentage frequency. The study used the χ2 test (categorical variables), the independent t-test (continuous, normally distributed variables) and the Mann-Whitney U (non-normally distributed variables) for comparisons between the groups. The r-Pearson and Spearman correlations were used for assessing the association between continuous variables. Survival curves and Cox proportional hazard model (univariate and multivariate) were used to determine the predictors for longer TTF/OS during ABI/ENZA treatment. A level of *p*<0.05 was recognized as statistically significant.

## 3 Results

### 3.1 Characteristics of the study group (overall population)

A summary of the basic characteristics of the patients is presented in **Table 1**.

**Table 1:** Clinicopathological characteristics of the study group.

Drug n (%)	All 420 (100)	ABI 320 (76.2)	ENZA 100 (23.8)	p-value ABI vs ENZA
Age				
n=	420	320	100	
Median (IQR), years	69 (64-75)	69 (64 – 76)	69 (63.5-74)	0.679
< 70 years. n (%)	215 (51.2)	141 (50.3)	54 (54)	0.520
≥ 70 years. n (%)	205 (48.8)	159 (49.7)	46 (46)	
Gleason scale				
n=	381	286	95	
Median (IQR)	8 (7-9)	-	–	<0.001*
≥8. n (%)	216 (51.4)	147 (45.94)	69 (69)	
<8. n (%)	165 (39.3)	139 (43.44)	26 (26)	
Missing data. n (%)	39 (9.3)	34 (10.62)	5 (5)	
ECOG				
n=	402	306	96	
0. n (%)	81 (19.3)	50 (15.63)	31 (31)	0.001*
1. n (%)	321 (76.4)	256 (80.0)	65 (65)	
Missing data. n (%)	18 (4.3)	14 (4.37)	4 (4)	
Treatment approach at PCa diagnosis				
n =	412	312	100	0.672
Radical. n (%)	164 (39)	126 (39.37)	38 (38)	
Palliative. n (%)	248 (59.1)	186 (58.13)	62 (62)	
Missing data. n (%)	8 (1.9)	8 (2.5)	–	
Site of metastases				
n=	413	315	98	
Bone only. n (%)	195 (46.43)	138 (43.13)	57 (57)	0.013
Visceral ± bone. n (%)	218 (51.90)	177 (55.31)	41 (41)	
Missing data. n (%)	7 (1.67)	5 (1.56)	2 (2)	
PSA ng/mL median (IQR)	107 (31.6 – 309.9)	130.1 (38.4 – 362.7)	59.5 (16.9 – 181)	0.002*
Type of ADT				
Bilateral orchidectomy. n (%)	19 (4.5)	13 (4.1)	6 (6.0)	0.984
LHRH agonists. n (%)	378 (90)	291 (90.9)	87 (87.0)	
LHRH antagonists. n (%)	15 (3.6)	10 (3.1)	5 (5.0)	
Missing data. n (%)	8 (1.9)	6 (1.9)	2 (2.0)	
Docetaxel treatment				
n=	404	306	95	
For mHSPC. n (%)	122 (29.1)	94 (29.38)	28 (28)	0.818
For mCRPC. n (%)	279 (66.4)	212 (66.25)	67 (67)	
Missing data. n (%)	19 (4.5)	14 (4.37)	5 (5)	
Type of progression at inclusion				
n=	418	318	100	
Biochemical. n (%)	82 (19.52)	66 (20.63)	16 (16)	0.105
Radiological. n (%)	41 (9.76)	23 (7.19)	18 (18)	
Both. n (%)	295 (70.24)	229 (71.56)	66 (66)	
Missing data. n (%)	2 (0.48)	2 (0.62)	–	
Median year of treatment	-	2017	2019	
BMI kg/m^2^				
n=	410	310	100	
median (IQR)	28.1 (25.4-31.3)	28.1 (25-31.1)	28.1 (25-31.9)	0.332
**≥**25. n (%)	314 (74,8)	240 (75)	74 (74)	
<25. n (%)	96 (22.8)	70 (21.87)	26 (26)	
Missing data. n (%)	10 (2.4)	10 (3.13)	–	
≥50% PSA decline				
*n*=	343	255	88	
Yes. n (%)	220 (52.38)	143 (44.69)	77 (77)	<0.001*
No. n (%)	123 (29.29)	112 (35)	11 (11)	
Missing data. n (%)	77 (18.33)	65 (20.31)	12 (12)	
Total DXL dose				
*n*=	339	259	80	
≥750 mg. n (%)	176 (41.90)	126 (39.38)	50 (50)	0.030*
< 750 mg. n (%)	163 (38.81)	133 (41.56)	30 (30)	
Missing data. n (%)	81 (19.29)	61 (19.06)	20 (20)	
PSA nadir during or after DXL treatment				
*n*=	336	248	88	
≥17.35 ng/mL. n (%)	168 (40)	141 (44.06)	27 (27)	<0.001*
<17.35 ng/mL. n (%)	168 (40)	107 (33.44)	61 (61)	
Missing data. n (%)	84 (20)	72 (22.5)	12 (12)	

ABI, Abiraterone acetate; ADT, androgen deprivation therapy; BMI, body mass index; DXL, docetaxel; ECOG, Eastern Cooperative Oncology Group; ENZA, enzalutamide; IQR, interquartile range; mCRPC, metastatic castration-resistant prostate cancer; mHSPC, metastatic hormone-sensitive prostate cancer; PCa, prostate cance; LHRH, a luteinizing-hormone-releasing hormone; PSA, prostate-specific antigen, *- statistically significant.

The study enrolled 420 patients who met inclusion criteria and were treated with ABI or ENZA. 72.6% of patients were treated with ABI and 23.8% with ENZA. Most of the patients were younger (51.2%) than 70 years. The median GS was 8, ECOG 1 (76.4%). In 59.1% of cases, PCa was diagnosed in the disseminated stage of disease, in 46.4% with the bone-only confined disease. DXL was the most commonly used in the mCRPC stadium of the disease (66.4%). The median PSA was 107 ng/mL at the start of ABI/ENZA treatment. The most common type of ADT was treatment with LHRH agonists (90%). Most enrolled patients were qualified for the program based on the biochemical and radiological progression (70.4%). Median PSA nadir during or after DXL was 17.35 ng/mL (IQR 3.43 – 76.69 ng/mL). The median DXL dose was 750 mg (IQR 510 – 990).

ABI and ENZA populations were statistically different in terms of several clinicopathologic factors, i.e. grading, pattern of metastases, and ECOG. In the ENZA group, PCa was less differentiated (≥8; 72.6 vs. 51.4; *p*<0.001), metastases occurred more frequently in the bone than in the viscera (56.2 vs. 41.8%; *p*=0.013), and patient performance status was better in the ENZA group (ECOG 0 32.3 vs. 16.3; *p*=0.001). A statistical comparison of the ABI and ENZA groups is shown in **Table 1**.

### 3.2 Analysis of TTF in the study group

**Figure 1:**
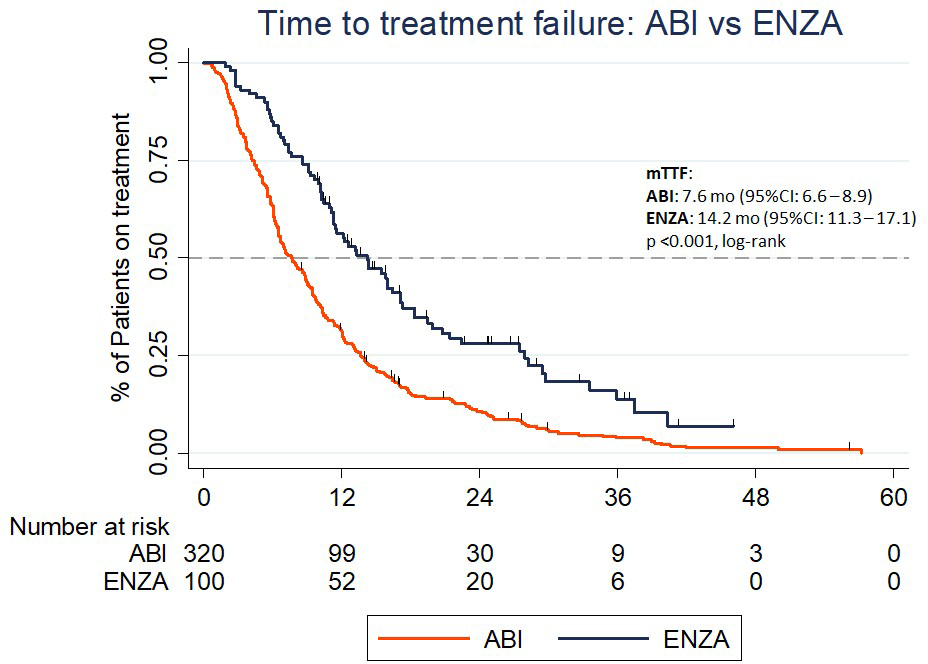
The time to treatment failure in the Abiraterone acetate and Enzalutamide group.

Median TTF for the overall population was 9.2 months (95% CI: 8.0 – 10.1). Median TTF in the ENZA group was statistically longer than in the ABI group (14.2 vs. 7.6 mo.; *p*<0.001; **Figure 1**). Median TTF was longer in ECOG 0 patients vs. ECOG 1 (11.3 vs. 8.8 mo.; *p*=0.021), BMI ≥25 vs. BMI <25 (9.5 vs. 6.5 mo.; *p*=0.009). Patients who were qualified for ABI/ENZA therapy with concurrent biochemical and radiological progression had shorter median TTF than patients who experienced the single type of progression (8.8 vs. 10.8 mo.; *p*<0.001).

DXL treatment in metastatic hormone-sensitive prostate cancer (mHSPC) vs. mCRPC did not affect TTF (*p*=0.743). However, patients who received ≥750 mg of DXL in total had longer median TTF (9.6 vs. 7.7 mo.; *p*=0.015). Patients with PSA nadir <17.35 ng/mL during or after DXL treatment had longer median TTF versus those who did not reach PSA levels below that value (12.2 vs. 6.5 mo., respectively; *p*<0.001). All analyzed variables are presented in **Table 2**. In the time-subgroup analysis (treatment between 2018 - 2021), the median TTF for both drugs was 10.6 mo. (95% CI: 9.2 – 12.0). TTF of ABI and ENZA was 7.6 mo (95% CI: 6.2 – 9.5) and 14.2 mo (95% CI: 11.3 -17.1), respectively (p <0.001).

**Table 2:** Predictive factors determining the time to treatment failure (univariate and multivariate analysis).

Variable	Median TTF (months)	HR	95% CI	*p*-value
A. Univariate analysis				
**Drug type**				
ABI (ref.)	7.6	–	–	–
ENZA	14.2	0.51	0.39 – 0.66	<0.001*
ECOG				
0 (ref.)	11.3	–	–	–
1	8.8	1.37	1.05 – 1.79	0.021*
BMI				
<25	6.5	–	–	–
≥25	9.5	0.73	0.57 – 0.92	0.009*
Gleason score				
<8 (ref.)	10.3	–	–	–
≥8	7.9	1.18	0.95 - 1.46	0.128
Age				
≥ 70 (ref.)	9.3	–	–	
<70	9.0	0.96	0.78- 1.17	0.685
Primary stage				
I-III (ref.)	9.4	–	–	–
IV)	9.1	1.10	0.89 - 1.35	0.386
Castration method				
Surgical (ref.)	9.6	–	–	–
Pharmacological	8.9	1.02	0.64- 1.65	0.916
Location of metastases				
Bone only (ref.)	8.9	–	–	–
Visceral ± bone	8.8	0.98	0.80 - 1.20	0.859
≥ 50% PSA decline				
No (ref.)	5.5	–	–	–
Yes	13.8	0.28	0.22 – 0.35	<0.001*
DXL therapy				
mHSPC (ref.)	8.8	–	–	–
mCRPC	9.3	1.04	0.83 - 1.30	0.743
Total DXL dose (mg)				
<750 (ref.)	7.7	–	–	–
≥750	9.6	0.75	0.60 - 0.95	0.015*
PSA nadir during or after DXL (ng/mL)				
<17.35 (ref.)	12.2	–	–	–
≥ 17.35	6.5	1.91	1.55 - 2.46	<0.001*
Initial progression type				
Radiological or PSA (ref.)	10.8	–	–	–
Both	8.8	1.57	1.25 – 1.99	< 0.001*
B. Multivaraite analysis				
Drug type				
ABI (ref.)	–	0.59	0.43 – 0.83	0.002*
ENZA				
ECOG				
0 (ref.)	–	1.11	0.81 - 1.53	0.520
1				
BMI				
<25 (ref.)	–	0.87	0.65 - 1.18	0.373
≥25				
Total DXL dose (mg)				
<750 (ref.)	–	0.87	0.66 – 1.14	0.317
≥750				
PSA nadir during or after DXL (ng/mL)				
<17.35 (ref.)	–	1.77	1.33 – 2.34	<0.001*
≥ 17.35				
Initial progression type				
Radiological or PSA (ref.)	–	1.33	0.99 – 1.79	0.057
Both				

ABI, abiraterone acetate; BMI, body mass index; CI, confidence interval; DXL, docetaxel; ECOG, Eastern Cooperative Oncology Group; ENZA, enzalutamide; HR, hazard ratio; mCRPC, metastatic castration-resistant prostate cancer; mHSPC, metastatic hormone-sensitive prostate cancer; PSA, prostate-specific antigen; ref, reference value; TTF, time to treatment failure; *- statistically significant.

In multivariate analysis, ENZA treatment (*p*=0.002) and PSA nadir during or after DXL treatment < 17.35 ng/mL (*p*<0.001) were related to statistically longer TTF (**Table 2**).

### 3.3 Analysis of OS in the study group

**Figure 2:**
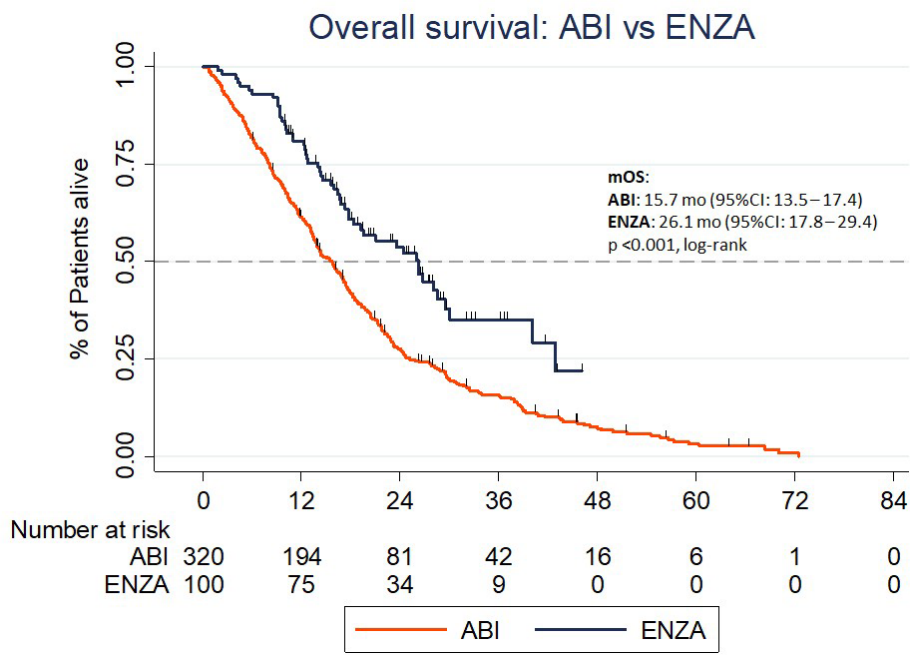
The overall survival in the Abiraterone acetate and Enzalutamide group.

For the whole population, the median OS was 17 months (95% CI:15.6-18.3). Median OS for patients treated with ENZA (26.1 mo.; 95% CI: 17.8-29.4) was significantly longer than for treatment with ABI (15.7, 95% CI 13.5 - 17.4; *p*<0.001, **Figure 2**). In univariate analysis, treatment with ENZA (*p*<0.001), ECOG 0 (*p*=0.014), BMI ≥25 (*p*=0.002), eligible for treatment due to one type of progression vs. multiple (*p*=0.002), treatment with DXL of at least 750 mg in total (*p*=0.001), ≥50% PSA response (*p*<0.001) during ABI or ENZA and PSA nadir < 17.35 during or after DXL treatment (*p*<0.001) were associated with longer median OS (**Table 3**). There was a positive, non-significant trend in longer median OS in patients who were primarily diagnosed with early-stage cancer (*p*=0.054). In patients treated between 2018 and 2021, the population’s median OS was 19.5 mo (95% CI: 16.7 – 22.8). The mOS in the ABI group was 16.7 mo (95% CI: 13.2 – 20.4). The median OS in the ENZA group was 26.1 mo (95% CI: 17.7 – 24.9). The difference was statistically significant (p = 0.001).

**Table 3:** Prognostic factors determining the overall survival (univariate and multivariate analysis).

Variable	Median OS (months)	HR	95% CI	*p*-value
A. Univariate analysis				
Drug type				
ABI (ref.)	15.7	–	–	–
ENZA	26.1	0.54	0.40 - 0.73	<0.001*
ECOG				
0 (ref.)	22.5	–	–	–
1	16.1	1.45	1.08 - 1.95	.014*
BMI				
<25	12.7	–	–	–
≥25	17.8	0.67	0.52 - .86	0.002*
Gleason score				
<8 (ref.)	18.7	–	–	–
≥8	14.3	1.17	0.93 - 1.47	0.181
Age				
≥ 70 (ref.)	17.7	–	–	–
<70	16.1	1.04	0.84 - 1.29	0.716
Primary stage				
I-III (ref.)	18.7	–	–	–
IV	15.9	1.24	0.99 - 1.55	0.054
Castration method				
Surgical (ref.)	20	–	–	–
Pharmacological	16.8	0.88	0.54 - 1.43	0.597
Location of metastases				
Bone only (ref.)	17.6	–	–	–
Visceral ± bone	16.3	1.00	0.81 - 1.24	0.968
≥ 50% PSA decline				
No (ref.)	10.5	–	–	–
Yes	23.3	0.37	0.29 - 0.47	<0.001*
DXL therapy				
mHSPC (ref.)	16.3	–	–	–
mCRPC	17.6	0.91	0.72 - 1.16	0.459
Total DXL dose (mg)				
<750 (ref.)	15.8	–	–	–
≥750	18.4	0.66	0.52 – 0.84	0.001*
PSA nadir during or after DXL (ng/mL)				
<17.35 (ref.)	22.5	–	–	–
≥ 17.35	14.3	1.89	1.48 – 2.42	<0.001*
Initial progression type				
Radiological or PSA (ref.)	20.3	–	–	–
Both	16.1	1.46	1.15 - 1.86	0.002*
B. Multivaraite analysis				
Drug type				
ABI (ref.)	–	0.62	0.42 – 0.93	0.019*
ENZA				
ECOG				
0 (ref.)	–	1.22	0.85 - 1.75	0.270
1				
BMI				
<25 (ref.)	–	0.92	0.66 - 1.28	0.610
≥25				
Primary stage				
I-III (ref.)	–	1.17	0.88 – 1.54	0.276
IV				
Total DXL dose (mg)				
<750 (ref.)	–	0.71	0.53 – 0.96	0.027*
≥750				
PSA nadir during or after DXL (ng/mL)				
<17.35 (ref.)	–	1.56	1.15 – 2.16	0.004*
≥ 17.35				
Initial progression type				
Radiological or PSA (ref.)	–	1.16	0.85 - 1.59	0.350
Both				

ABI, abiraterone acetate; BMI, body mass index; CI, confidence interval; DXL, docetaxel; ECOG, Eastern Cooperative Oncology Group; ENZA, enzalutamide; mCRPC, metastatic castration-resistant prostate cancer; mHSPC, metastatic hormone-sensitive prostate cancer; OS, overall survival; PSA, prostate-specific antigen; ref, reference value; *- statistically significant.

In multivariate analysis, ENZA treatment (*p*=0.019), total DXL dose >750 mg in total (*p*=0.027) and PSA nadir <17.35 during or after DXL treatment (*p*=0.004) were associated with longer OS (**Table 3**).

### 3.4 PSA response during ABI/ENZA

The 50% PSA decline was higher in the ENZA group than in the ABI group (87.5% vs. 56%; *p*<0.001). Patients who experienced ≥ 50% PSA decline during ABI/ENZA had statistically longer TTF in comparison with men who had <50% PSA decline (13.9 vs. 5.6 mo., respectively; *p*<0.001, log-rank). Median OS was also longer in men with ≥ 50% PSA decline (23.3 vs. 10.5 mo.; *p*<0.001, log-rank).

### 3.5 Treatment after progression

After progression on ABI or ENZA, 75/166 patients (45.18%) were treated with a subsequent line of therapy (ABI: 50 patients, 41.3%; ENZA: 25 patients, 55.5%). The patients were treated with DXL rechallenge, cabazitaxel, radium-223 dichloride, ABI/ENZA and clinical trials. The OS for patients treated with at least one subsequent line of therapy was 21.2 months (95% CI: 16.56 - 26.15), and the median of OS for patients who were not treated was 11 months (95% CI 7.57 - 14.61). There were no statistical differences in the number of subsequent therapy lines between ABI and ENZA (*p*=0.101).

## 4 Discussion

Real-life studies allow for a better understanding of disease courses in a specific population of patients and facilitate the selection of a drug, which is especially important for practicing oncologists. The choice of the drug by clinicians may be based on patient comorbidities, expected side effects, patient preferences and cost-effectiveness. The multicenter retrospective analysis in the current study is the first study to describe the outcomes for mCRPC patients treated with ABI and ENZA in Poland. The study was conducted on all subsequent patients from these centers who met the criteria for participation in the Polish drug program, which meets the criteria of a retrospective case-control study (evidence level IIIE). There is a lack of randomized, comparative phase III trials in patients treated with ENZA and ABI in mCRPC, which justifies the current real-life clinical data analysis.

COU-AA-301 and AFFIRM were registered trials that determined the role of ABI and ENZA in mCRPC patients pretreated with DXL chemotherapy (7, 8). A total of 800 patients were treated with ENZA, and 300 were treated with a placebo in AFFIRM. The final analysis revealed that the median of OS was 18.4 vs. 13.6 months, respectively (p < 0.001, HR: 0.63; 95% CI: 0.53–0.75). The trial also met the secondary endpoints, including the proportion of patients with a reduction in the PSA level by 50% or more (54% vs. 2%, *p<*0.001), the time to PSA progression (8.3 vs. 3.0 mo.; *p*<0.001), radiographic progression-free survival (rPFS) (8.3 vs. 2.9 mo.; *p*<0.001). The analysis of HR for death showed the superiority of ENZA over placebo in all patient subgroups, including age (65< vs. ≥65 years), baseline ECOG, type of progression at entry study, visceral disease and PSA level at baseline (7). In the COU-AA-301 trial, 797 patients were treated with ABI and prednisone, and 398 received a placebo. The median OS for the study group was 15.8 months (95% CI: 14.8-17) vs. 11.2 months (10.4-13.1; HR 0.74, 95% CI: 0.64-0.86; *p*<0·0001). The trial also met the secondary endpoints: median time to PSA progression (8.5 vs. 6.6 mo. *p*<0·0001), median rPFS (5.6 vs. 3.6 mo. *p*<0.0001), and proportion of patients who had a PSA response (29.5% vs. 5.5%; *p*<0.0001). The analysis of HR also confirmed the superiority of the study drug over the placebo in subgroups, including age, ECOG and type of progression (8).

In the current study, the median OS was 15.7 months in the ABI group (95% CI: 13.5 - 17.4) and 26.1 months (95% CI: 17.8-29.4) in the ENZA group. The difference between drugs was statistically significant. TTF was also longer in the ENZA group (14.2; 95% CI: 11.3-17.6 mo.) than the ABI group (7.6; 95% CI: 6.64-8.85; *p*<0.001). The 50% PSA decline was higher in the ENZA group than in the ABI group (87.5 vs. 56%; *p*<0.001), which was higher than in the registered trials (56 vs. 29.5%). The 50% PSA decline was observed more frequently in the ENZA group (87.5%) than in the ABI group (56%; *p*<0.001). The result of OS in the current study and the registered trial was comparable to the ABI group (15.7 vs. 15.8 months) but differed from the ENZA group (26.1 vs. 18.4 months). However, the length of treatment was longer in the ENZA group (*p*<0.001), TTF and rPFS are not comparable oncological outcome measures.

The observational study of real-life clinical data was analyzed in many countries, and results vary between the different populations of patients. The main differences between the studies include the number of patients, the line of treatment (pre or post-chemotherapy) and the following lines of treatment after progression.

The most extensive observational study was published by Schoen et al. and presented the results of treatment on 5,822 US veterans. It shows that the OS was longer in patients treated with ENZA than ABI (24.2 vs 22.1 months) in pre- and post-chemotherapy treated patients. Importantly, patients with cardiovascular diseases also had better survival (9). Also, a direct comparison of drugs in 10,308 chemotherapy-naive patients with CRPC based on the 2014-2018 French population study showed that patients treated with ENZA had a better OS than ABI (34.2 vs. 31.7 mo.) (10). Li et al. also recently published the results of a retrospective cohort population-based study in the unselected Taiwanese population, which showed that treatment with ENZA (n=118) was associated with better OS than treatment with ABI (n=1046), although without differences in TTF (11). In another unselected Australian population of 250 patients (53% of ABI; 38% of ENZA post-chemo), patients treated with ENZA had a greater PSA response (70.3% vs. 39.5%). The OS was longer in the ENZA group, 29 months (95% CI: 21.3-36.7) vs. 7 months (95% CI: 0-18.5%; *p*=0.002) in the ABI group. The authors did not find any factors significantly associated with OS, including the Gleason scale (12). Interesting data come from Chowdhury et al., who analyzed the oncological outcomes among patients from 16 countries with mCRPC treated in the first line of therapy with ABI, ENZA or chemotherapy. The OS for ABI and ENZA was the same (27.1 months) (13).

Data from selected post-DXL-treated patients show similar results. Contrary to the pre-DXL setting, in post-DXL, the OS in the ENZA (26 ± 7 (12.3-39.7)) was longer than the ABI (13 ± 1.6 (9.8-16.2); 0.021) cohort of patients. Moreover, the rPFS was longer in the ENZA (11± 5.1 (1.1-20.9) than the ABI (pre-and post-DXL) group. PSA ≥50% decline occurred more frequently in the ENZA group than in the ABI group (*p*=0.02). The authors concluded that the good prognostic factors for rPFS were ENZA treatment, age ≥75 years and PSA ≥50% decline at 12 weeks of treatment. The authors did not find any prognostic factors for OS (14).

Another Taiwanese study that indirectly compared the ENZA (*n*=13) and ABI (*n*=63) in post-DXL chemotherapy showed that the OS from second-line hormone treatment was 30.2 months in the ABI group and 16.2 months in the ENZA group, although statistical significance was not shown. The same study shows no difference between PSA and PFS responses. PSA 50% response was seen in 48.4% in ABI and 69.2% in the ENZA group (*p*=0.171); the median PFS was 7.3 months (95% CI: 4.79-9.80) in ABI and 9.5 months (95%CI: 5.743-13.257) in ENZA (p of log rank=0.766) (15). In Austrian populations of patients, the OS for ABI was 14 months (mean: 15.8 ± 0.9 months), and for ENZA it was 19 months (mean: 17.2 ± 1.4 months). A randomized phase II cross-over study confirmed that ENZA is associated with better biochemical response, however, without changes in time to PSA progression (16). Hu et al. found a difference in OS between ENZA and ABI (17.9 vs. 15.4 mo.; *p*=0.8224) (17). Other studies show different OS in post-DXL treated with ABI. In the Marret et al. trial, the OS was 13.4 months, although only 58 patients were analyzed (18).

Finally, Wei et al. performed a meta-analysis of 5,199 patients treated with ABI and ENZA in randomized clinical trials. Contrary to rPFS and time to PSA progression, which were significantly better in the ENZA group, the mOS did not vary significantly between ABI and ENZA (HR=1.03, 95% CI: 0.854-1.242) (19). The same results regarding OS came from Bianchi et al. analysis (20). Another indirect analysis of AFFIRM and COU-AA-301 trials favors ENZA in terms of time to PSA progression, PSA response and radiological PFS, although without difference in OS (21). Chung et al. also studied the effectiveness of both drugs in sequential treatment. The sequence ABI-ENZA is better regarding oncological outcomes than ENZA-ABI (22). In Poland, sequential treatment is not reimbursed.

The current study attempted to identify the prognostic factors associated with ABI and ENZA therapy. Although the univariate analysis revealed that ENZA treatment, ECOG 0, BMI ≥25 kg/m^2^, total DXL dose ≥750 mg, ≥50% PSA decline, and PSA nadir during or after DXL treatment <17.35 ng/mL are factors associated with better survival, the multivariate analysis shows that ENZA treatment and total DXL dose ≥750 mg and PSA nadir <17.35 ng/mL during or after DXL treatment were independent prognostic factors for longer OS. Early PSA response is a good independent prognostic factor in next-generation androgen receptor inhibitors (23, 24). The ≥50% drop in PSA from baseline within the three months of treatment correlated with better OS and PFS (23). However, although it was found that DXL dose has an impact on the prognosis of patients with PCa, treatment with an early DXL was not found to be a prognostic factor. Patients with hormone-sensitive PCa were not included in clinical trials, and data in such a population of patients are limited and not well explored. Additionally, it was studied if BMI affects prognosis during antiandrogen treatment. The majority of patients (74.4%) were overweight or obese. In univariate analysis, patients with BMI ≥ 25 kg/m^2^ had longer TTF and OS, although this lacked statistical significance in multivariate analysis. Other studies suggest that obesity may play a protective role associated with increased survival (25–27).

In COU-AA-301, the authors of the study found that patients who received DXL for more than three months had better OS than those treated for less than three months (8). Chi et al. published a risk model for predicting OS in chemotherapy-pretreated patients treated with ABI, including ECOG 2, presence of liver metastases and time from ADT to the start of ABI ≤36 months (28). The multivariate analysis of the hazard ratio for death showed that ECOG 0, mean pain score on Brief Pain Inventory-Short Form <4, PSA progression at study entry, and no visceral disease at screening were associated with better survival (7). Patients with age > 75, Charlson comorbidity scores > 2, presence of symptoms, time from prostate cancer diagnosis < 3 years and time from last chemotherapy < 6 months had lower survival (17). Multivariate analysis revealed that PSA response, Gleason score ≥8 and PSA-doubling time <2 months correlated with OS. Patients with visceral metastases had worse oncological outcomes in terms of OS (2.8 vs. 18; *p*=0.0007) and PFS (2.8 vs. 6.8; *p*=0.0088) (29). Another study showed that low levels of miR-21 are an unfavorable prognostic factor in PCa patients (30).

In the current study, it was shown that ENZA treatment may be related to more favorable oncological outcomes than ABI treatment in the Polish population of patients. There was no difference in the efficacy of ABI and ENZA on time to treatment failure in docetaxel-naive and docetaxel-pretreated prostate cancer patients. A 50% decline in PSA is an indicator of longer TTF and OS. Due to the non-randomized and retrospective nature of the analysis, the current results require prospective validation. Although many different observational and metanalyses show that treatment with enzalutamide has a better treatment outcome, there is no single explanation for the results. The main difference between drugs includes different action mechanisms because abiraterone inhibits 17α-hydroxylase/C17,20-lyase (CYP17), and enzalutamide has a threefold mechanism of action. First, it is a potent, competitive binder of androgens at the level of the androgen receptor (AR), and it prevents the translocation of the AR from the cytoplasm to the nucleus. Within the nucleus, it inhibits AR binding to chromosomal DNA, which prevents further transcription of tumor genes. Therefore, compared to abiraterone, enzalutamide may act more selectively and comprehensively on the AR signaling pathway in prostate cancer cells (31). The indirect analysis of drugs has several limitations which may also affect the results. One of the major disadvantages of real-life data trials is the heterogenicity of the groups and follow-up time in the study. In the current cohort of patients, the differences included the ECOG scale, GS and location of metastases. The population of patients treated with ENZA was in better performance status (ECOG 0: 32.3 vs. 16.3%), had a higher percent of bone-only limited metastases (58.2 vs. 43.8%), received the higher cumulative dose of DXL (62.5 vs. 48.7%), and more patients had PSA nadir of <17.35 during or after DXL (69.3% vs 43.1%) but had less differentiated tumors (72.6 vs. 51.4%) which may interfere with the results. ABI was introduced earlier than ENZA in Poland, which limits the adjustment of drug selection. Patients included in our study were not treated during the same period (ABI 2014-2021, ENZA 2018-2021); however, the subgroup analysis of patients treated during the same time supports the superiority of ENZA in terms of TTP and OS. The unbalanced population of patients were also an issue in similar studies. The current study limitations include an unbalanced study population, the retrospective nature of the study and the lack of some clinical aspects like pain score or quality of life (similar to other published studies).

The current study confirmed the clinical activity of ABI and ENZA in the Polish population of patients. The presented analysis suggests that treatment with ENZA may be related to more favorable outcomes than treatment with ABI.

## Data availability statement

The raw data supporting the conclusions of this article will be made available by the authors, without undue reservation.

## Ethics statement

The studies involving human participants were reviewed and approved by local bioethics committee in Olsztyn (5/21/VII.). Written informed consent for participation was not required for this study in accordance with the national legislation and the institutional requirements.

## Author contributions

Conceptualization, DS and MW; methodology, DS and MW; statistical analysis, MW; writing—original draft preparation, DS, data collection: all the authors, supervision: IS. All authors contributed to the article and approved the submitted version.
